# Pretreatment neutrophil-to-lymphocyte ratio and its dynamic changes are associated with the overall survival in advanced cancer patients undergoing palliative care

**DOI:** 10.1038/srep31394

**Published:** 2016-08-11

**Authors:** Weiwei Zhao, Zhenyu Wu, Yintao Li, Huixun Jia, Menglei Chen, Xiaoli Gu, Minghui Liu, Zhe Zhang, Peng Wang, Wenwu Cheng

**Affiliations:** 1 Department of Integrated Therapy, Fudan University Shanghai Cancer Center, Shanghai, China; 2 Department of Oncology, Shanghai Medical College, Fudan University, Shanghai, China; 3 Department of Biostatistics, School of Public Health, Key Laboratory of Public Health Safety, Ministry of Education, Fudan University, Shanghai, China; 4 Department of Oncology, Shandong Cancer Hospital, Shandong Academy of Medical Sciences, Jinan, China; 5 Department of Integrative Oncology, Fudan University Shanghai Cancer Center, Shanghai, China

## Abstract

The objective of this study was to investigate the prognostic value of pretreatment NLR and its dynamic changes responsive to palliative care in advanced cancer patients. The study was retrospectively assessed in 378 consecutive advanced cancer patients receiving palliative care, and in an extended follow-up study of 106 of those patients. The cutoff value of pretreatment NLR was determined to be 3.0. In the 378 advanced cancer patients, 89 had pretreatment NLR ≤ 3, and 289 had an NLR > 3. Univariate and multivariate analyses showed that tumor stage, palliative care, albumin level, and pretreatment NLR (HR: 1.514, 95% CI: 1.125~2.038, *P* = 0.006) were independent prognostic indicators of OS. Moreover, in the follow-up cohort of 106 readmitted patients, 43 patients achieved a decreased NLR after palliative care, while the remaining 63 patients showed an increased NLR. Univariate and multivariate analyses showed that an increase in NLR was significantly associated with a poor survival (HR: 2.506, 95% CI: 1.474~4.261, *P* = 0.001). In conclusion, pretreatment NLR and changes in NLR independently predicted OS in advanced cancer patients undergoing palliative care. Incorporating NLR into clinical practice may better inform the prognosis and therapy decisions of advanced cancer patients in palliative settings.

Despite the evident progress achieved in cancer therapies, the need for palliative care in individuals has increased. Palliative care has been recommended as an essential component throughout the cancer trajectory that can be delivered concurrently with standard anticancer treatment^1^,^2^. Patients with advanced cancer usually have a reduced quality of life and a shorter survival, while early integration of palliative care might improve quality of life and survival for a wide range of advanced cancers^3^. As a poor prognosis may be associated with a lower quality of life and a higher rate of clinical complications throughout the remaining lifetime of advanced cancer patients, survival estimations are essential for proper treatment in palliative care^4^. Accurate predictions of the prognosis are needed in advanced cancer patients because they may help physicians’ clinical decision-making regarding cancer therapies and care planning and help patients and their families prepare for the time ahead^5^,^6^. However, estimates of individual survival time are difficult to make, and tools to evaluate the progression of advanced cancer patients are scarce^4^. Clinicians’ predictions of survival in patients with advanced cancer are often overly optimistic and not reliable, with an accuracy ranged from 32% to 39% in different settings^7^,^8^. As a result, the identification of a simple and objective prognostic indicator for advanced cancer patients is needed to better guide clinical practice.

Cancer-related inflammation has been recognized as one of the hallmarks of cancer, with a vital role in the modulation of the tumor microenvironment^9^. Multiple lines of evidence indicate that inflammation plays an important role in carcinogenesis and tumor progression by fostering cancer cell proliferation, promoting tumor angiogenesis, invasiveness and metastasis, and affecting tumor response to systemic therapies^10^. Cancer-related inflammation results in poor survival, and inflammation-related factors in cancer are predictors of outcomes in various cancers^11^,^12^. The neutrophil/lymphocyte ratio (NLR), a marker of host inflammation, has been found to be an independent prognostic factor of adverse outcomes in various cancers, such as gastrointestinal cancer, liver cancer, renal cancer, lung cancer, and ovarian cancer^13^. Nevertheless, studies on the association between NLR and survival in advanced cancer patients in palliative settings are limited.

Therefore, this study was designed to investigate the clinical influence of elevated NLR levels on survival in advanced cancer patients in palliative settings, and to investigate the value of changes in NLR (increased or decreased) after palliative care as a prognostic indicator of OS.

## Results

### Patient characteristics

**Figure 1:**
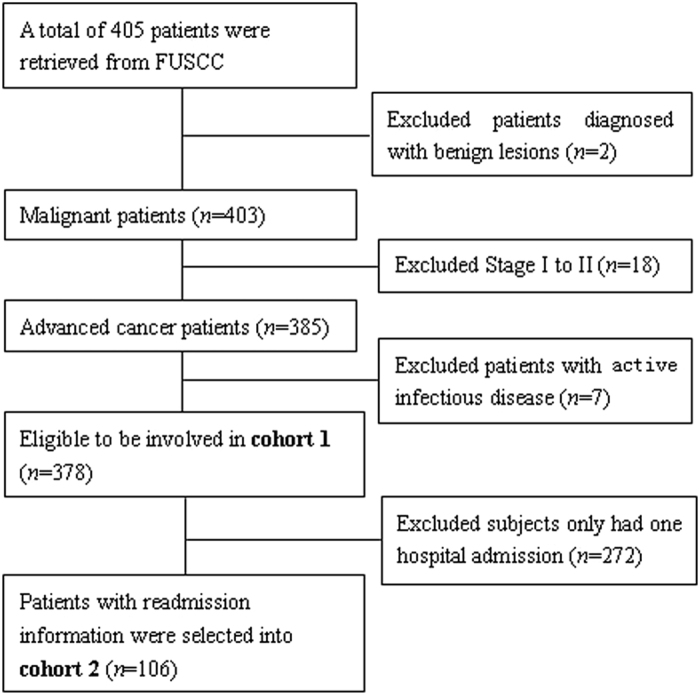
Flow chart of patients excluded from the study.

A total of 405 patients were retrieved from the database. Then, 2 patients diagnosed with benign lesions, 18 patients with early tumor stages (stage I or II), and 7 patients with active infectious diseases were excluded. Thus, 378 eligible advanced cancer patients (stage III and IV) were identified in cohort 1 in the study. Of the 378 patients, 106 with readmission information were selected in cohort 2. The study cohort flow diagram is shown in Fig. 1.

In cohort 1, 209 patients were male (55.3%), and 169 patients were female (44.7%). The median age of the entire cohort was 64 years old (ranging from 14 to 94 years). There were 23 patients who had stage III tumors (6.1%) and 355 patients who had tumors in stage IV (93.9%). The patients were distributed to gastrointestinal tumors (198/378), thoracic cancer (86/378), urogenital cancer (59/378), head and neck neoplasms (16/378), and other tumors (19/378) according to the primary tumor site. Ninety-nine patients received palliative chemoradiotherapy (89 patients received palliative chemotherapy and 10 patients received palliative radiotherapy), while the remaining 279 patients received best supportive care. No one in our study received palliative operation within 1 month. The median NLR value was 5.5 (IQR: 3.37~9.66). The median duration of follow-up of the entire cohort was 445 days (range, 1~882 days). In cohort 2, 56 patients were male (52.8%), and 50 patients were female (47.2%). The median age of this cohort was 63 years (ranging from 29 to 88 years). Thirteen patients had tumors in stage III (12.3%), and 93 patients had stage IV tumors (87.7%). Sixty patients (56.6%) with gastrointestinal tumor accounted for the vast majority of the cohort. The median change in NLR was 0.561 (IQR: −1.004~3.386). The median duration of follow-up of these participants was 509 days (range, 28~882 days).

### Determination of the NLR cutoff value

Converting a continuous variable into a binary one is frequently performed in clinical studies. Patients are simply categorized into groups of “High” and “Low”, which may establish the criteria for diagnosing or predicting prognosis. However, no acknowledged clinical cutoff values were available for NLR in advanced cancer patients^13^, a RCS analysis could be used to determine objective cutoff points^14^,^15^. Figure S1 depicts the relationship between pretreatment NLR (in logarithmic form) and the log relative hazard using a smoothing spline curve. The blue dotted line (1.08) indicates a turning point of the log relative hazard; thus, we assumed that a baseline NLR of 3 (Exp(1.08) approximately equals 3) was a potential cutoff value for advanced cancer patients following palliative care. Accordingly, patients with pretreatment NLR greater than 3 were categorized into a high NLR (HNLR) group and patients with pretreatment NLR less than or equal to 3 were categorized into a low NLR (LNLR) group.

### Association of NLR and NLR changes with clinicopathological features

The pretreatment clinicopathological features of patients from cohort 1 are shown in Table 1. The proportion of LNLR was 23.5% (89/378) and of HNLR 76.5% (289/378). As a continuous variable, the NLRs were significantly higher in males (*P* = 0.029), in patients with best supportive care (*P* < 0.001), in patients with a smoking history (*P* = 0.013) and in patients with an abnormal nutritional status (*P* = 0.004). As a dichotomous variable, there were no significant differences between the LNLR and HNLR group in comparisons of those clinicopathological characteristics (*P* > 0.05).

In cohort 2, we first calculated changes in NLR and categorized patients into a decreased group (NLR change < 0) and an increased group (NLR change > 0). The differences in NLR changes were compared regarding clinicopathological features as a continuous variable. The NLR of the second admission was significantly lower than that of the first admission in patients with tumor stage III compared with patients in stage IV (*P* = 0.009). The NLR of the second admission was significantly higher in the best supportive care group than in the palliative chemoradiotherapy group (*P* = 0.001). There were 43 patients whose NLR decreased and 63 patients in whom it increased after palliative care when compared with the first hospital admission. From a proportion point of view, 76.92% (10/13) of the patients in stage III had a decreased NLR, which was significantly higher than that of patients in stage IV (33/93, 35.48%) (*P* = 0.004). Besides, the proportion of increased NLR was significantly higher in patients receiving best supportive care than in patients receiving palliative chemoradiotherapy (*P* < 0.001). No other statistically significant different proportions were observed between the decreased and increased NLR groups (Table 2).

### Associations between OS and NLR and NLR changes

**Figure 2:**
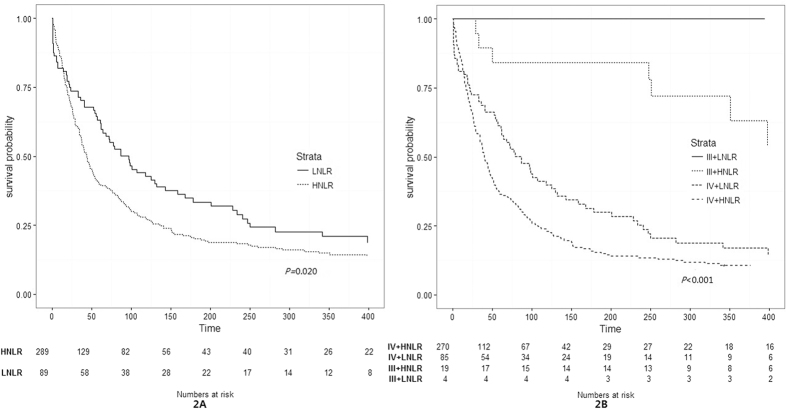
Overall survival of patients with palliative care stratified by pretreatment NLR; tumor stage and NLR (cohort 1). NLR: neutrophil~to~lymphocyte ratio; LNLR: low NLR (pretreatment NLR ≤ 3); HNLR: high NLR (pretreatment NLR > 3).

In cohort 1, patients were divided into an HNLR and an LNLR group, the median survival time was 44 (95% CI: 38~52) days and 97 (95% CI: 62~132) days, respectively. According to the Kaplan-Meier analysis, the HNLR group was significantly associated with a worse OS than the LNLR group (*P* = 0.020) (Fig. 2A). The univariate survival analysis showed that OS was significantly associated with tumor stage (HR: 5.441, 95% CI: 2.687~11.020, *P* < 0.001), primary tumor site (HR: 0.880, 95% CI: 0.792~0.977, *P* = 0.016), palliative care (HR: 0.432, 95% CI: 0.328~0.569, *P* < 0.001), nutritional status (HR: 1.732, 95% CI: 1.336~2.246, *P* < 0.001), albumin level (HR: 0.943, 95% CI: 0.926~0.960, *P* < 0.001) and pretreatment NLR (HR: 1.377, 95% CI: 1.049~1.806, *P* = 0.020) (Table 3). Multivariable Cox proportional hazard models also revealed that tumor stage, palliative care, albumin level and NLR were independent prognostic factors of OS. After controlling for tumor stage, primary tumor site, palliative care, nutritional status, and albumin level, NLR (HNLR vs. LNLR) remained significantly (HR: 1.514, 95% CI: 1.125~2.038, *P* = 0.006) associated with OS (Table 3). We hypothesized that high pretreatment NLR and more advanced disease (stage IV) would be associated with worse survival. Further analyses were conducted to compare OS stratified by tumor stage and pretreatment NLR. As expected, no subjects in the stage III + LNLR group died during the study period. Patients in stage III + HNLR and stage IV + LNLR groups showed significantly better OS than the IV + HNLR group (*P* < 0.001 and *P* = 0.013, respectively) (Table 4 and Fig. 2B).

**Figure 3:**
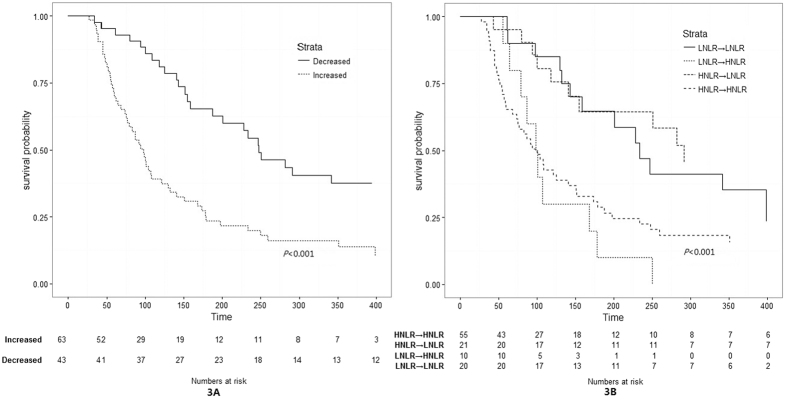
Overall survival of patients with palliative care stratified by NLR change (cohort 2). LNLR: low NLR (pretreatment NLR ≤ 3); HNLR: high NLR (pretreatment NLR > 3).

In cohort 2, patients were divided into an increased and a decreased group, and the median survival time was 98 (95% CI: 75~121) days and 248 (95% CI: 188~409) days, respectively. Both univariate and adjusted multivariate analyses showed that an increased NLR had a poor OS (HR: 2.609, 95% CI: 1.638~4.154, *P* < 0.001 and HR: 2.506, 95% CI: 1.474~4.261, *P* = 0.001, respectively) (Table 5). We hypothesized that patients with a pretreatment LNLR that became a HNLR would have the worst OS. Further subgroup analyses were performed on associations of NLR change with OS. In the univariate analysis, hazard ratios of patients with a pretreatment LNLR or HNLR that become HNLR after palliative care, were approximately 3 and 2 times, respectively, to that of LNLR patients. When demographic and disease-specific factors were adjusted for, more significant results were obtained (Table 5). The Kaplan-Meier analyses confirmed that the increased NLR group had a worse OS than the decreased NLR group (*P* < 0.001) (Fig. 3A); the subgroup K-M curves were depicted in Fig. 3B.

## Discussion

This study demonstrated that the pretreatment NLR was significantly and negatively associated with overall survival in advanced cancer patients. The finding remained significant after controlling for sociodemographic characteristics and disease features. In addition, after palliative care, patients with a decreased NLR showed improved outcomes. These results suggest that NLR may serve as an independent prognostic indicator of OS and could be used as a monitoring index for the therapeutic effects of palliative care on advanced cancer patients during tumor follow-up.

Survival prediction is a persisting challenge in the area of cancer, especially in the advanced cancer population. Accurate survival estimation would be helpful for clinical decision making, care planning, patients and their families’ psychosocial and social preparation^5^,^6^. Clinician predictions of survival are the most commonly used approach to form a prognosis^7^. The approach includes temporal, surprise and probabilistic questions that are all subjective. The temporal question “How long will this patient live?” is answered as a specific time frame (eg, 3 days, 6 months). It is often not specified and possibly results in confusion among health care professionals and patients with a low accuracy of 10–40%^7^,^16^. The surprise question “Would I be surprised if this patient died in (specific time frame)?” is posed to the healthcare professional. The answer is binominal (yes or no), while the threshold for “surprise” may vary according to each healthcare professional. As demonstrated by previous studies, the positive predictive value ranges from 30% to 84% and the negative predictive value ranges from 69% to 97%^17^,^18^. The high false-positive rates limit its role in definitive prognosis prediction. The probabilistic question “What is the probability of survival within a specific time frame?” tends to be more accurate than the temporal question. The accuracy of probabilistic predictions is on the order of 33–100%^16^. However, it is also a subjective and time frame dependent question that requires interpretation^7^. Established actuarial estimation of survival are classified as prognostic factors (including decreased performance status, delirium, dysphagia, cancer anorexia–cachexia, dyspnea, inflammation, malnutrition, and phase angle) and prognostic models (including the Palliative Prognostic Score, Palliative Performance Scale, Palliative Prognostic Index, and Glasgow Prognostic Score)^7^. They are often portrayed as objective and may improve the accuracy of survival estimation. However, the interpretation of the symptoms mentioned above may vary with different clinicians, which limits its utility to a certain extent^19^. Prognostic models are also complicated because they require a comprehensive disease assessment and hematologic assay. Therefore, more accurate and easier to obtain prediction tools for survival prognostication are warranted.

The NLR represents an easily measured, reproducible, inexpensive, and objective marker of systemic inflammation in clinical practice. Mounting evidence has demonstrated the association between high NLR and negative survival outcomes in a variety of cancers, including gastrointestinal cancer, liver cancer, renal cancer, lung cancer, and ovarian cancer^13^. However, its potential clinical and practical value in advanced cancer patients receiving palliative care regarding pretreatment risk stratification and the medical decision-making process remains unclear. Our analysis expands upon previous studies by analyzing a new cohort of advanced cancer patients in a palliative setting and an extended follow-up cohort to evaluate the relationship between NLR and survival outcomes. To date, the acknowledged cutoff value for the clinical application of NLR has not been established, varying from 2.0 to 5.0 in heterogeneous studies^13^. Therefore, a RCS regression was used to explore the nonlinear relationship of NLR and hazard ratio in advanced cancer patients. The result showed that the NLR value of 3, which had been most employed in previous studies^13^, could be used as a cutoff value for predicting OS. Then, we evaluated the predictive value of NLR in survival in advanced cancer patients receiving palliative care. The NLR was significantly higher in male patients, in patients receiving best supportive care, and in patients with smoking history or abnormal nutritional status. This finding is consistent with data from Viers *et al.* and Shimizu *et al.*^20^,^21^. Furthermore, we demonstrated that patients with a high NLR had a significantly shorter OS compared to low NLR cancer patients (*P* = 0.020). In the multivariate analysis, high NLR was a significant independent predictive factor of poor OS (*P* = 0.006). It is widely accepted that tumor stage and albumin level are strong and independent prognostic factors in cancer patients^21^,^22^. In the current study, tumor stage and albumin level (*P* < 0.001) were also shown to be independent prognostic factors of decreased OS. Besides, we also found that patients receiving best supportive care had a worse outcome (*P* = 0.002). We further stratified patients into different tumor stage groups to assess the confounding effect of tumor stage on NLR. In this study, the predictive value of NLR was significant in both stage III cancer patients and stage IV cancer patients (*P* < 0.001). Our results demonstrated that NLR can independently predict OS in both stage III and stage IV advanced cancer patients in a palliative setting.

The role of pretreatment NLR has been investigated in various cancers, but the predictive role of its dynamic changes has not been adequately explored and remains controversial. Wang F *et al.*, Lee *et al.*, Ohno *et al.*, and Luo G *et al.* found that reductions in NLR levels were associated with improved survival in different cancer patients^23^,^24^,^25^,^26^. However, Formica *et al.* observed that those in whom NLR was increased or maintained had a 67% reduction in risk of death compared to patients with a significant NLR decrease, which seemed to affect survival differently^27^. In the current study, we demonstrated that an increase in NLR from low to high levels resulted in a significantly shorter OS compared with those with a decrease in NLR from high to low levels (*P* < 0.001). The median survival in the decreased NLR group was 5 months longer than in the increased NLR group. In the multivariate analysis, increased NLR was independently associated with poorer OS (HR: 2.506, *P* = 0.001). Furthermore, the HRs of patients with elevated NLR after palliative care and stable high NLR were approximately 3 and 2 times, respectively, compared to patients with stable low NLR before and after palliative care. These results indicate that an elevated NLR after palliative care was a strong prognostic indicator for patients in the palliative setting, regardless of the pretreatment NLR. These results suggest that pretreatment value and dynamic changes of NLR can discriminate the advanced cancer patients into two different groups with good or poor outcome. In the group with poor outcome, we may only be able to offer the best supportive care to patients, and we must take a more active role in helping the patients and their families preparing for death. However, in the good outcome group, we can choose some less toxic anti-cancer agents or palliative radiotherapy for patients to help alleviate their symptoms, improve quality of life, and extend survival under the permissibility of physical condition. Besides, the therapeutic options may be adjusted to the dynamic change of NLR.

Up to date, only 4 studies have investigated the association between pre-treatment NLR and survival in cancer patients receiving palliative care and they all showed that the pre-treatment NLR was prognostically important^28^,^29^,^30^,^31^. However, unlike the existing 4 studies, whose study population was terminal cancer patients, we investigated the association in advanced cancer patients (including stage III and stage IV) as palliative care was integrated earlier in the course of advanced cancer in our country. Second, in these 4 studies, patients with active infections which may cause changes in the hematological variables were not excluded. Third, the influences of different palliative care options or different types of cancers on NLR or the survival were not accounted for. Last, they do not explore the relationship of NLR change with OS.

The mechanism between high NLR and poor patient outcomes may be related to the cancer-related inflammation, which has been recognized as a hallmark of cancer that substantially contributes to the development and progression of malignancies^9^. The cancer-related inflammation encompasses tumor-derived and host-derived cytokines, small inflammatory proteins, and infiltrating immune cells acting in the local tumor microenvironment, which is stimulated by highly proliferative tumor^32^. There is substantial cross-talk between the mediators and cytokines in the local tumor microenvironment and systemic circulation, promoting myelopoiesis and contributing to a blockade of myeloid cell maturation. The tumor-derived secretome can condition distant sites, such as the bone marrow and spleen, to increase myelopoiesis. Cancer myelopoiesis is associated with defective myeloid-cell differentiation, resulting in the accumulation and persistence of immature myeloid cells, such as MDSCs, in the circulation^33^. Circulating granulocytes are likewise increased during cancer-mediated myelopoiesis, with neutrophils the most abundant, which account for most circulating white cells. Ordinarily, neutrophils are not released from the bone marrow until mature; however, in the context of inflammation, the neutrophil precursors-myelocytes and promyelocytes-might be released. The prognostic and predictive usefulness of circulating neutrophils is apparent as an independent measure or as part of the NLR^13^. Treatment for those patients is becoming increasingly aggressive, mostly documented by an increasing use of palliative chemoradiotherapy^34^. However, our study found that a patient whose pretreatment NLR is higher than 3 may not need such intensive treatment to prolong one’s survival. While for the patients whose NLR changed from high to low, more aggressive treatment strategy may be a better choice.

This study investigated for the first time the association between pretreatment NLR and change in NLR and OS in advanced cancer patients receiving palliative care. However, our study had some limitations. First, the data were collected at a single tertiary care center with a relatively small sample that was entirely of Chinese origin, which may limit the generalizability of the results. Second, because of the retrospective, nonrandomized nature of the study, firm conclusions cannot be drawn regarding the relationship between the NLR index and patient outcomes. Further research should be conducted, which we hope these findings can stimulate.

In conclusion, our study provides the important finding that NLR is an independent predictor of survival in advanced cancer patients receiving palliative care. Given the ease of its calculation from routinely measured blood test without additional costs, the NLR could result in clinical translational advances in the identification of high-risk cancer patients, which could aid in clinical decision making.

## Methods

### Data source and study cohort

The palliative care unit in Fudan University Shanghai Cancer Center (FUSCC), Shanghai, China, was established in 2006 to provide symptom management and, psychosocial and social support for cancer patients. Patients treated at the palliative care unit of FUSCC between July 2013 and October 2015 were retrospectively reviewed. Data regarding age, gender, smoking history (yes/no), family history (yes/no), concomitant disease (yes/no), nutritional status, hospital stay, primary tumor site (gastrointestinal tumor, thoracic cancer, urogenital cancer, head and neck neoplasm, and otherwise tumors), tumor stage, and albumin level were extracted from medical records. Peripheral blood cell tests including a differential white cell count were performed 1–3 days before palliative care. For each patient, these data were collected at the time of initiating palliative care. Clinical and demographic information were extracted from the medical data platform of FUSCC by trained staff using standardized data collection and quality-control procedures, resulting in reliable data for analysis. The research was approved by the institutional review board of FUSCC. Inform consent was granted a waiver due to the retrospective nature of the study. All treatments were performed in accordance with relevant guidelines and regulations.

Two cohorts of patients were identified in our study. In cohort 1, we examined the associations of several potential risk factors with OS. Patients with the following inclusion criteria were enrolled: (1) a hospitalization for palliative care; (2) the presence of various cancers confirmed by histopathology or at least cytology; (3) availability of pretreatment peripheral blood test results from 1–3 days prior to palliative care; and (4) availability of all clinical data. Patients with benign or early stage (I,II) tumors, and those with active infectious disease were excluded from the analysis. In cohort 2, we explored the relationship between changes in NLR after undergoing palliative care and OS. Patients in cohort 1 who had a second admission to our unit were enrolled. The above-mentioned demographic, disease-specific factors, and blood cell tests were also obtained at the second admission. The last follow-up date was in December, 2015. The NLR was derived from the quotient of the absolute neutrophil count and the absolute lymphocyte count. Abnormal nutritional status was defined as an unintentional weight loss >5% in the previous 3 months or a food intake below 75% of the normal requirement in the preceding week according to the ESPEN guidelines for nutrition screening^35^. Concomitant disease was defined as self-reported cardiac disease, hypertension, diabetes, or any cerebrovascular disease, that might be associated with systemic inflammation. The active infection was defined as patients with fever (>38 °C) accompanied by positive culture treated with antibiotic-directed therapy^20^,^36^. Overall survival (OS) time was defined as the period from the date of initial treatment in the palliative care unit of FUSCC to death or the last follow-up.

### Statistics Analysis

Categorical variables were described as totals and frequencies, comparisons were conducted using the chi-squared or Fisher’s exact test as appropriate. Continuous variables were described as medians and inter quartile ranges (IQR) and comparisons were made by Wilcoxon sum rank test or Kruskal-Wallis *H* test. For our research, a logarithmic transformation was applied to pretreatment NLR because of its skewed distribution (*P* < 0.001). A restricted cubic spline (RCS) function, which can be used to examine the relationship between continuous variables and the outcome of interest^14^,^15^, was used to assess the nonlinear associations between the pretreatment NLR and OS and to determine objective cutoff value. Survival curves were estimated by the Kaplan-Meier method and differences between groups were compared using the Log-rank test. Univariate and multivariable adjusted hazard ratios (HRs) and 95% confidence intervals (95% CIs) were calculated using Cox proportional hazards models, and to estimate the association of demographic and clinicopathologic variables of interest with OS. A two-sided *P* < 0.05 was considered statistically significant. Statistical analyses were performed using SAS 9.2 (Cary, NC, USA) and R software version 3.1.2 ( http://www.r-project.org) with the rms package.

## Additional Information

**How to cite this article**: Zhao, W. *et al.* Pretreatment neutrophil-to-lymphocyte ratio and its dynamic changes are associated with the overall survival in advanced cancer patients undergoing palliative care. *Sci. Rep.* **6**, 31394; doi: 10.1038/srep31394 (2016).

## Supplementary Material

Supplementary Information

## Figures and Tables

**Table 1:** Clinicopathological features of the included patients in cohort 1 (*N* = 378).

Clinicopathological features	*N*	NLR (continuous)	*P*	NLR (dichotomous)		*P*
		Median (IQR)		LNLR (*n* = 89)	HNLR (*n* = 289)	
Gender			0.029			0.130
Male	209	5.813 (3.667~10.50)		43 (48.31%)	166 (57.44%)	
Female	169	5.333 (3.091~8.889)		46 (51.69%)	123 (42.56%)	
Age			0.756			0.3070
<65	203	5.667 (3.273~9.833)		52 (58.43%)	151 (52.25%)	
≥65	175	5.500 (3.460~9.618)		37 (41.57%)	138 (47.75%)	
Tumor stage			0.054			0.473
III	23	3.833 (3.133~6.000)		4 (4.49%)	19 (6.57%)	
IV	355	5.667 (3.429~9.889)		85 (95.51%)	270 (93.43%)	
Primary tumor site			0.295			0.551
Gastrointestinal tumors	198	6.163 (3.696~10.50)		40 (44.94%)	158 (54.67%)	
Thoracic cancers	86	5.422 (3.273~9.091)		23 (25.84%)	63 (21.80%)	
Urogenital neoplasms	59	4.867 (3.050~7.571)		15 (16.85%)	44 (15.22%)	
Head and neck neoplasms	16	5.962 (2.640~9.778)		5 (5.62%)	11 (3.81%)	
Other tumors	19	4.417 (2.750~8.385)		6 (6.74%)	13 (4.50%)	
Palliative care			<0.001			0.196
PCR	99	4.364 (2.875~6.667)		28 (31.46%)	71 (24.57%)	
BSC	279	6.125 (3.583~10.73)		61 (68.54%)	218 (75.43%)	
Family history			0.979			0.109
No	264	5.333 (3.462~9.889)		58 (65.17%)	206 (71.28%)	
Yes	109	6.243 (3.111~9.200)		28 (31.46%)	81 (28.03%)	
Unknown	5	14.72 (10.44~19.00)		3 (3.37%)	2 (0.69%)	
Smoking history			0.013			0.301
No	265	5.250 (3.143~9.091)		68 (76.40%)	197 (68.17%)	
Yes	105	6.404 (3.778~10.63)		19 (21.35%)	86 (29.76%)	
Unknown	8	9.722 (7.517~11.63)		2 (2.25%)	6 (2.08%)	
Concomitant disease			0.850			0.311
No	229	5.558 (3.279~9.861)		58 (65.17%)	171 (59.17%)	
Yes	149	5.500 (3.533~9.429)		31 (34.83%)	118 (40.83%)	
Nutrient status			0.004			0.204
Normal	107	4.375 (2.800~7.333)		31 (34.83%)	76 (26.30%)	
Abnormal	268	6.021 (3.652~10.59)		57 (64.04%)	211 (73.01%)	
Unknown	3	3.778 (2.480~13.25)		1 (1.12%)	2 (0.69%)	
Hospital Stay			0.335			0.250
≤14 days	205	5.764 (3.458~10.83)		53 (59.55%)	152 (52.60%)	
>14 days	173	5.444 (3.308~9.091)		36 (40.45%)	137 (47.40%)	

NLR: neutrophil-to-lymphocyte ratio; IQR: inter quartile ranges; LNLR: low NLR (pretreatment NLR ≤ 3); HNLR: high NLR (pretreatment NLR > 3); PCR: palliative chemoradiotherapy; BSC: best supportive care.

**Table 2:** Clinicopathological features of the included patients in cohort 2 (*N* = 106).

Clinicopathological features	*N*	NLR change (continuous)	*P*	NLR change (dichotomous)		*P*
		Median (IQR)		Decreased (*n* = 43)	Increased (*n* = 63)	
Gender			0.735			0.611
Male	56	0.550 (−0.759~3.444)		24 (55.81%)	32 (50.79%)	
Female	50	0.572 (−1.209~2.500)		19 (44.19%)	31 (49.21%)	
Age			0.661			0.325
<65	63	0.550 (−1.180~3.518)		28 (65.12%)	35 (55.56%)	
≥65	43	0.667 (−0.381~2.500)		15 (34.88%)	28 (44.44%)	
Tumor stage			0.009			0.004
III	13	−1.569 (−2.548~−0.250)		10 (23.26%)	3 (4.76%)	
IV	93	0.950 (−0.667~3.518)		33 (76.74%)	60 (95.24%)	
Primary tumor site			0.147			0.609
Gastrointestinal tumors	60	1.115 (−0.718~4.000)		24 (55.81%)	36 (57.14%)	
Thoracic cancers	14	0.343 (−0.883~7.980)		5 (11.63%)	9 (14.29%)	
Urogenital neoplasms	23	−0.250 (−2.548~1.250)		12 (27.91%)	11 (17.46%)	
Head and neck neoplasms	6	2.212 (1.110~3.444)		1 (2.33%)	5 (7.94%)	
Other tumors	3	0.550 (−1.254~4.650)		1 (2.33%)	2 (3.17%)	
Palliative care			0.001			0.001
PCR	54	−0.315 (−1.832~1.486)		30 (69.77%)	24 (38.10%)	
BSC	52	1.740 (0.233~4.166)		13 (30.23%)	39 (61.90)	
Family history			0.467			0.148
No	73	0.521 (−1.068~3.171)		33 (76.74%)	40 (63.49%)	
Yes	33	0.773 (−0.374~3.518)		10 (23.26%)	23 (36.51%)	
Smoke history			0.812			0.994
No	74	0.619 (−0.900~2.500)		30 (69.77%)	44 (69.84%)	
Yes	32	0.485 (−1.276~3.842)		13 (30.23%)	19 (30.16%)	
Concomitant disease			0.825			0.531
No	63	0.572 (−0.900~3.444)		24 (55.81%)	39 (61.90%)	
Yes	43	0.533 (−1.254~2.702)		19 (44.19%)	24 (38.10%)	
Nutrient status			0.311			0.356
Normal	42	0.419 (−1.254~2.375)		20 (46.51%)	22 (34.92%)	
Abnormal	63	0.619 (−0.759~3.900)		23 (53.49%)	40 (63.49%)	
Unknown	1	2.702 (2.702~2.702)		0 (0.00%)	1 (1.59%)	
Hospital stay			0.051			0.323
≤14 days	53	0.233 (−1.209~2.375)		24 (55.81%)	29 (46.03%)	
>14 days	53	1.250 (−0.381~3.518)		19 (44.19%)	34 (53.97%)	

NLR: neutrophil-to-lymphocyte ratio; IQR: inter quartile ranges; PCR: palliative chemoradiotherapy; BSC: best supportive care.

**Table 3:** Prognostic factors associated with overall survival in cohort 1 using univariate and multivariable analyses (*N* = 378).

Prognostic factors	Univariate analyses		Multivariate analysis	
	Unadjusted HR (95% CI)	*P*	Adjusted HR (95% CI)	*P*
Gender (Female vs. Male)	1.038 (0.829~1.299)	0.746		
Age (≥65 vs. <65)	1.083 (0.866~1.355)	0.484		
Tumor stage (IV vs. III)	5.441 (2.687~11.020)	<0.001	5.154 (2.409~10.03)	<0.001
Primary tumor site	0.880 (0.792~0.977)	0.016	0.993 (0.893~1.104)	0.896
Palliative care (PCR vs. BSC)	0.432 (0.328~0.569)	<0.001	0.630 (0.468~0.847)	0.002
Family history (Yes vs. No)	1.174 (0.921~1.498)	0.195		
Smoke history (Yes vs. No)	0.856 (0.663~1.105)	0.233		
Nutrient (Abnormal vs. Normal)	1.732 (1.336~2.246)	<0.001	1.141 (0.866~1.502)	0.349
Concomitant disease (Yes vs. No)	1.016 (0.810~1.275)	0.891		
Hospital stay (≤14 vs. >14 days)	0.995 (0.794~1.246)	0.962		
Albumin level	0.943 (0.926~0.960)	<0.001	0.956 (0.937~0.976)	<0.001
NLR (HNLR vs. LNLR)	1.377 (1.049~1.806)	0.020	1.514 (1.125~2.038)	0.006

NLR: neutrophil-to-lymphocyte ratio; HR: hazard ratio; CI: confidence interval; PCR: palliative chemoradiotherapy; BSC: best supportive care. LNLR: low NLR (pretreatment NLR ≤ 3); HNLR: high NLR (pretreatment NLR > 3).

**Table 4:** Unadjusted and Adjusted HRs for overall survival stratified by tumor stage and pretreatment NLR in cohort 1 (*N* = 378).

Stage + NLR	Unadjusted			Adjusted*		
	HR	95% CI	*P*	HR	95% CI	*P*
IV + HNLR (*n* = 270)	Reference	~		Reference	~	
IV + LNLR (*n* = 85)	0.709	0.539~0.931	0.013	0.659	0.487~0.893	0.007
III + HNLR (*n* = 19)	0.212	0.104~0.430	<0.001	0.226	0.104~0.489	<0.001
III + LNLR (*n* = 4)#	—	—		—	—	

NLR: neutrophi-to-lymphocyte ratio; HR, hazard ratio; CI, confidence interval

^*^Cox regression model controlling for Age; Gender; Primary tumor site; Palliative care; Family history; Smoke history; Nutrient status; Concomitant disease; Hospital stay; Albumin level.

^#^No events in III + LNLR group.

**Table 5:** Unadjusted and adjusted HRs for overall survival stratified by NLR change in cohort 2 (*N* = 106).

Prognostic factors		Unadjusted HR (95% CI)	*P*	Adjusted HR* (95% CI)	*P*
NLR	Decreased	Reference		Reference	
	Increased	2.609 (1.638~4.154)	<0.001	2.506 (1.474~4.261)	0.001
NLR change	LNLR → LNLR	Reference		Reference	
	LNLR → HNLR	3.017 (1.306~6.973)	0.010	4.061 (1.632~10.10)	0.003
	HNLR → LNLR	0.759 (0.344~1.678)	0.496	0.939 (0.401~2.201)	0.886
	HNLR → HNLR	2.237 (1.208~4.141)	0.010	2.936 (1.530~5.634)	0.001

NLR: neutrophil-to-lymphocyte ratio; HR, hazard ratio; CI, confidence interval.

^*^Cox regression model controlling for Age; Gender; Tumor stage; Primary tumor site; Palliative care; Family history; Smoke history; Nutrient status; Concomitant disease; Hospital stay; Albumin level.
