# Supplementary material for: Pretreatment neutrophil-to-lymphocyte ratio and its dynamic changes are associated with the overall survival in advanced cancer patients undergoing palliative care
Source: Sci Rep. 2016 Aug 11;6:31394. doi: 10.1038/srep31394 (PMC4980771; doi:10.1038/srep31394)
Supplement: Supplementary Information [file srep31394-s1.pdf]

Pretreatment neutrophil-to-lymphocyte ratio and its dynamic changes are associated with the overall survival in advanced cancer patients undergoing palliative care

Weiwei Zhao[1,2,*], Zhenyu Wu[3,*], Yintao Li[4], Huixun Jia[2], Menglei Chen[1,2], Xiaoli Gu[1,2], Minghui Liu[1,2], Zhe Zhang[1,2], Peng Wang[2,5,#], Wenwu Cheng[1,2,#]

[1]Department of Integrated Therapy, Fudan University Shanghai Cancer Center, Shanghai, China;
[2]Department of Oncology, Shanghai Medical College, Fudan University, Shanghai, China;
[3]Department of Biostatistics, School of Public Health, Key Laboratory of Public Health Safety, Ministry of Education, Fudan University, Shanghai, China;
[4]Department of Oncology, Shandong Cancer Hospital, Shandong Academy of Medical Sciences, Jinan, China;
[5]Department of Integrative Oncology, Fudan University Shanghai Cancer Center, Shanghai, China

Correspondence and requests for materials should be addressed to W.C. (e-mail: cwwxxm@sina.com) or P.W. (e-mail: wangp413@163.com)

* Weiwei Zhao and Zhenyu Wu contributed equally to this work.

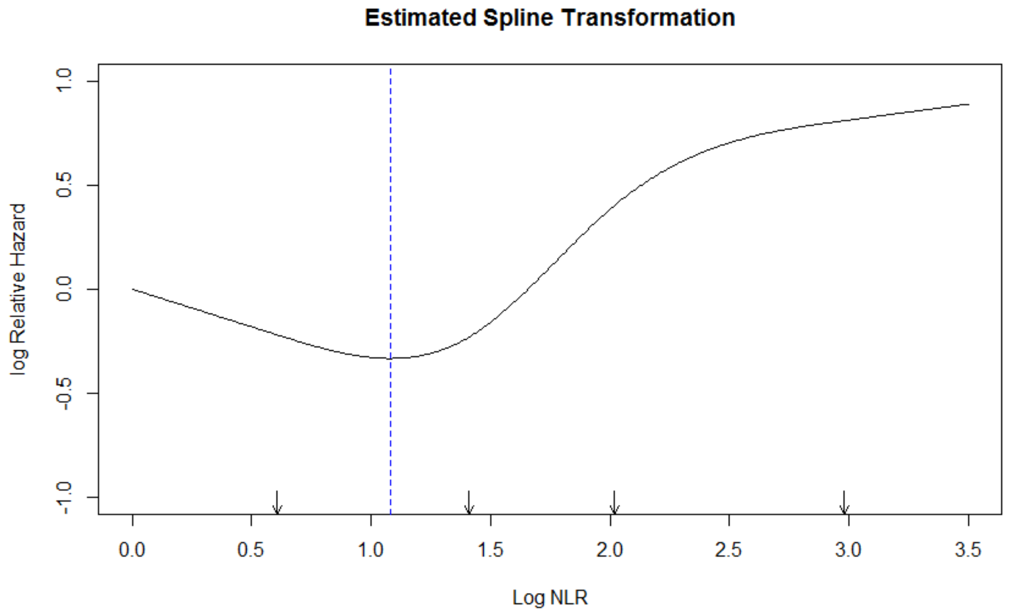

Figure S1. Relationship between the pretreatment NLR (in logarithmic form) and Log Relative Hazard based on RCS function with 4 knots.

NLR: neutrophil-to-lymphocyte ratio; RCS: restricted cubic spline
